# Natural Antisense Transcripts at the Interface between Host Genome and Mobile Genetic Elements

**DOI:** 10.3389/fmicb.2017.02292

**Published:** 2017-11-20

**Authors:** Hany S. Zinad, Inas Natasya, Andreas Werner

**Affiliations:** RNA Interest Group, Institute for Cell and Molecular Biosciences, Newcastle University, Newcastle upon Tyne, United Kingdom

**Keywords:** natural antisense transcripts, gene expression regulation, double stranded RNA (dsRNA), non-coding RNA, RNA interference, DNA methylation, histone modifications

## Abstract

Non-coding RNAs are involved in epigenetic processes, playing a role in the regulation of gene expression at the transcriptional and post-transcriptional levels. A particular group of ncRNA are natural antisense transcripts (NATs); these are transcribed in the opposite direction to protein coding transcripts and are widespread in eukaryotes. Their abundance, evidence of phylogenetic conservation and an increasing number of well-characterized examples of antisense-mediated gene regulation are indicative of essential biological roles of NATs. There is evidence to suggest that they interfere with their corresponding sense transcript to elicit concordant and discordant regulation. The main mechanisms involved include transcriptional interference as well as dsRNA formation. Sense–antisense hybrid formation can trigger RNA interference, RNA editing or protein kinase R. However, the exact molecular mechanisms elicited by NATs in the context of these regulatory roles are currently poorly understood. Several examples confirm that ectopic expression of antisense transcripts trigger epigenetic silencing of the related sense transcript. Genomic approaches suggest that the antisense transcriptome carries a broader biological significance which goes beyond the physiological regulation of the directly related sense transcripts. Because NATs show evidence of conservation we speculate that they played a role in evolution, with early eukaryotes gaining selective advantage through the regulatory effects. With the surge of genome and transcriptome sequencing projects, there is promise of a more comprehensive understanding of the biological role of NATs and the regulatory mechanisms involved.

## Introduction

**FIGURE 1:**
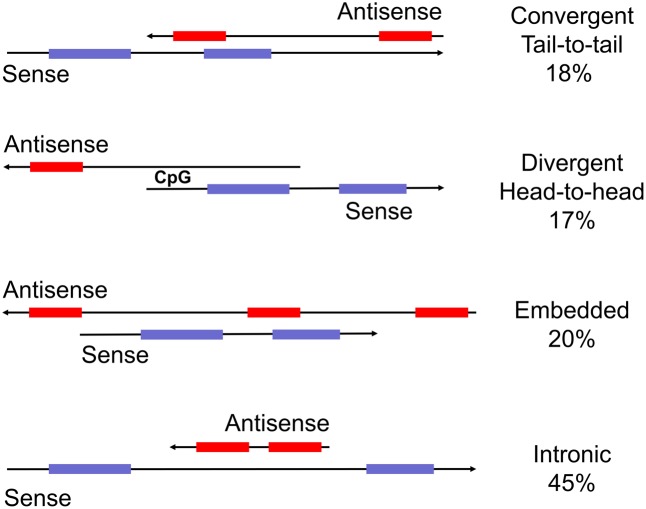
Schematic representation of sense and antisense transcripts from bi-directionally transcribed loci. Commonly used descriptions are head-to-head, tail-to-tail, embedded and intronic (from top). The percent representation in the human genome is from Balbin et al. (2015). Importantly, transcripts in tail-to-tail and full overlap configuration potentially form double-stranded RNA whereas the other two categories do not. Other terms used are ‘convergent’ (tail-to-tail), divergent (head-to-head), or ‘non-overlapping’ (embedded).

Natural antisense transcripts (NATs) are arguably the oldest group within the family of non-coding RNAs. The first examples of bi-directionally transcribed genes were detected as early as in the 1980s (Beiter et al., 2009). It then emerged that human and mouse imprinted gene clusters express antisense transcripts. Interestingly, antisense transcription is associated with allele-specific gene silencing, not only in imprinted gene clusters but also other bi-directionally transcribed loci (Verona et al., 2003; Carlile et al., 2009; Werner and Swan, 2010). The general and widespread expression of NATs emerged at the beginning of the genomic era with the computational analyses of human and mouse NATs (Lehner et al., 2002; Shendure and Church, 2002). These reports analyzed the ever increasing repository of full-length sequences and sequence tags for complementary transcripts and identified over a 100 sense–antisense pairs. They set the stage for a series of seminal computational and large scale experiments to detect complementary transcripts and decipher their putative biological roles (Kiyosawa et al., 2003; Chen et al., 2004; Katayama et al., 2005; Werner et al., 2007). The initial efforts to characterize antisense RNAs preceded the development of RNA-seq platforms and, as a consequence, only include reasonably abundant, stable and mostly cloned transcripts. Antisense transcripts were then defined as long, non-coding RNAs that are complementary to a coding transcript from the opposite strand (**Figure 1**). Nowadays, NATs are widely recognized as versatile regulators of gene expression. Intriguingly, many of the associated regulatory pathways involve double-stranded RNA intermediates that are reminiscent of viral structures or transposon intermediates.

Natural antisense transcription has been detected in bacteria, yeast and all eukaryotes. Extensive research into various aspects of RNA biology and epigenetics have revealed a variety of species-specific mechanisms dealing with bi-directional transcription and complementary RNA molecules. As a result, in multicellular organisms such as plants, *Caenorhabditis elegans* or mammals, NATs will trigger different mechanisms and elicit drastically different cellular responses. In plants, best described in *Arabidopsis thaliana*, complementary RNA triggers a strong RNA interference response and the formation of siRNAs from the double-stranded sequence (Baulcombe, 2004). Moreover, DNA methylation can be induced as a consequence of double stranded RNA (dsRNA) formation and the action of an RNA-dependent RNA polymerase (RdRP) (Matzke and Birchler, 2005). The system is thought to protect plants from viral infections and genomic parasites. *C. elegans* also expresses an RdRP and has the potential to amplify a dsRNA response that leads to endo-siRNA production from endogenous dsRNA structures (Gu et al., 2009). These are thought to enable self-recognition and prevent the integration of foreign DNA into the genome. More complex animals lack an amplifying system for dsRNA and, as a consequence, at least mechanisms that involve sense–antisense transcript hybridization will differ significantly between various organisms. To what extent natural antisense related dsRNA formation triggers an antiviral response, RNA interference or helps to control transposon activity is intensely debated. It appears that in chordates, including human and mouse, the response to dsRNA varies fundamentally between germ cells, stem cells and differentiated somatic cells (Cullen et al., 2013).

In recent years the field has focused on the characterization of specific sense–antisense transcript pairs predominantly in a pathophysiological context. The scope of this article is to discuss a few prominent examples of gene regulation by NATs and set them in context with evolutionary considerations.

## Mobile Genetic Elements and Antisense Transcription

The significance of NATs in a genomic context cannot be appreciated without considering the impact of mobile genetic elements, including transposons and viruses. For example, antisense transcription is often initiated by the insertion of transposable elements with promoter activity downstream of protein coding genes (Conley et al., 2008). NATs have also been associated with controlling the activity of transposons and mitigating the consequences of their insertion into a complex genome (Stein et al., 2015). Importantly, NATs that are co-expressed with their cognate sense transcripts may form dsRNA intermediates reminiscent of viral structures that activate an immune response, which in turn induces significant expression changes in the antisense transcriptome (Ilott et al., 2014).

The expansion of mobile genetic elements has not only increased genetic plasticity but also introduced promoters and enhancers to initiate the transcription of novel loci. Since the number of protein coding genes has not increased significantly during the evolution of complex organisms the transposition of genetic elements has primarily resulted in enhanced transcription of non-coding, regulatory RNAs (Mattick, 2001). This also applies to NATs, demonstrated by a moderate but significant accumulation of antisense transcriptional start sites downstream of protein coding genes. These coincide with ancient MIR and L2 transposon sequences; the observation that ancient transposons drive more antisense transcripts than the younger L1 or Alu elements suggests phylogenetic functional conservation of antisense transcription (Conley et al., 2008). Interestingly, the mammalian X chromosome shows an inverse trend: NATs are significantly under-represented despite an accumulation of transposable elements and a proposed role for L1 elements in maternal X chromosome inactivation (Emerson et al., 2004; Abrusan et al., 2008). Considering the potential of NATs to epigenetically silence the related protein-coding sense gene, it is conceivable that a bi-directional arrangement is detrimental in a monoallelic context whereas it may prove advantageous in a bi-allelic background (Werner et al., 2009).

**FIGURE 2:**
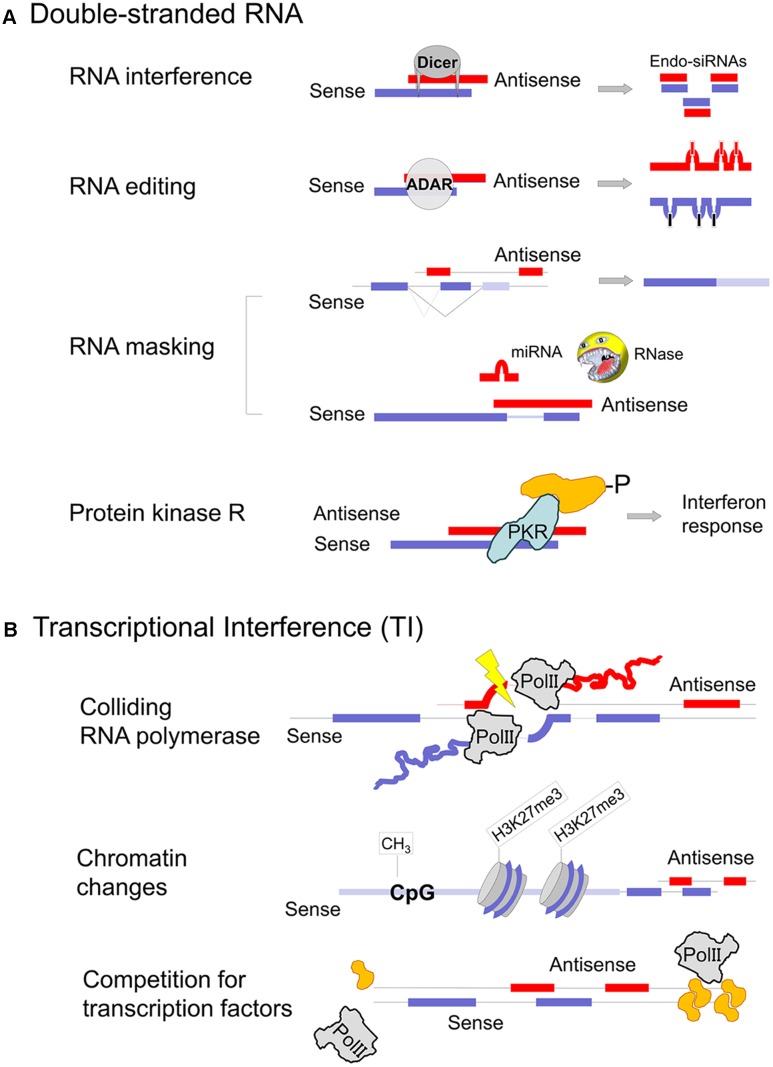
Mechanisms of gene regulation involving natural antisense transcripts. **(A)** Co-expression of sense and antisense transcripts in the same cell may cause dsRNA formation. RNA masking can have inhibitory as well as stimulatory consequences on the protein-coding sense mRNA expression, depending on the motif that is obstructed. Due to potential PKR activation dsRNA formation must either occur in specific cellular compartments or in specific cell types that do not rise an interferon response (germ cells and stem cells). Parts of the figure are modified from Wight and Werner (2013). **(B)** Transcriptional interference, where the expression of one transcript affects transcription of the opposite strand, can occur at several levels. Sense–antisense expression shows a discordant pattern, the majority as a result of antisense transcription induced chromatin changes (Weinberg and Morris, 2016).

Transposon mobilization, viral infection and sense/antisense expression can all form dsRNA intermediates that are potentially damaging to the cell. In vertebrates, two principal mechanisms have evolved to mitigate the deleterious consequences of transposon mobilization and protect cells from viral infections: the piRNA/endo-siRNA system and protein kinase R/interferon, respectively (**Figure 2A**). The two protective mechanisms show distinct expression patterns. In pluripotent stem cells, during early embryogenesis as well as in female and male germ cells the piRNA/endo-siRNA system restricts retro-transposition (Okamura and Lai, 2008). On the other hand, PKR/IFN are predominantly active in differentiated, somatic cells and provide protection against viral infections. To what extent RNA interference plays a role in somatic cells against viruses is a matter of intense debate (Cullen et al., 2013). Experimental attempts to demonstrate virus-derived siRNAs after infection of cultured cells are technically challenging and may not represent a physiologically relevant model (Jeffrey et al., 2017; tenOever, 2017). Accordingly, we found no evidence of abundant endo-siRNA expression in human cells, though the few loci that produced endo-siRNAs tended to be bi-directionally transcribed (Werner et al., 2014).

On the other hand, endo-siRNAs and piRNA are readily detectable in germ and zygotic cells. Interestingly, the short RNA pattern is qualitatively very similar between zygote and oocytes but distinctly different in spermatozoa where it includes piRNAs and endo-siRNAs from loci that potentially form dsRNA precursors (Garcia-Lopez et al., 2014). This may reflect pervasive transcription and the resulting highly complex transcriptome in these cells (Laiho et al., 2013; Soumillon et al., 2013). This assumption concurs with findings from Dicer knock-out mice that showed spermatogenic defects that coincided chronologically with transcriptional silencing and chromatin condensation (Korhonen et al., 2011). Moreover, abundant endo-siRNAs map to protein coding genes and potentially regulate the expression of their targets (Song et al., 2011). The finding that endo-siRNAs silence L1 retrotransposons through DNA methylation suggest that these short RNAs are in fact capable of establishing a widespread, cell specific genomic imprint in male germ cells (Chen et al., 2012). This observation has prompted speculations that spermatogenic endo-siRNAs and hence NATs could play an essential role in the evolution of complex organisms (Werner et al., 2015). The genome undergoes various changes during spermatogenesis such as demethylation and potential activation of transposable elements as well as genomic recombination that requires extensive DNA repair. We recently proposed a hypothesis how NATs help to detect genes that produce inadequate RNA output thus providing a genomic quality control (Werner et al., 2015). In various contexts RNA is being used to maintain genome integrity (Duharcourt et al., 2009) or distinguish self from novel genetic material (Gu et al., 2009). An RNA-based control mechanism to maintain integrity of the genome seems therefore conceivable.

Natural antisense transcripts are involved in regulating gene expression in an immune challenge; however, a protective reaction involving NATs seems unlikely. Conversely, recent evidence suggests that herpesviruses induce wide-spread host antisense transcription to interfere with the expression of pro-apoptotic genes (Wyler et al., 2017). Upon lipopolysaccharide exposure, human monocytes differentially express more than 200 long non-coding RNAs of which about half can be categorized as NATs (Ilott et al., 2014). Two of these were further characterized and shown to regulate the proinflammatory mediators IL1β (Interleukin 1β) and CXCL8 (C-X-C Motif Chemokine Ligand 8). Likewise, the expression of IL1α (Interleukin 1 α) is regulated by a NAT (Chan et al., 2015). These findings indicate that NATs in immune cells have specific, gene regulatory tasks whereas in germ cells a broader role in genome maintenance has been suggested.

## Functional Relevance of NATs

The contribution of NATs to maintaining cellular homeostasis is a matter of intense scrutiny – though many questions remain. On the one hand NATs are abundant in every genome and numbers tend to increase in higher eukaryotes. On the other hand, the evidence of antisense transcripts contributing to *homeostatic* gene regulation – apart from parentally imprinted genes – is circumstantial.

Genome-wide studies have established phylogenetic conservation, expression pattern or co-regulation with other transcripts and support the biological relevance of non-coding transcripts, as do loss of function experiments (Diederichs, 2014; Goff and Rinn, 2015). The phylogenetic conservation of NATs has been scrutinized widely and the perception has changed over time. The observation in early computational experiments that antisense transcripts show splicing differences and often minimal sequence identity between closely related species argued against stringent conservation (Veeramachaneni et al., 2004; Wood et al., 2013). However, recent reports based on microarray or RNAseq data, taking conserved transcription or expression patterns into account, confirm phylogenetic conservation of antisense transcription (Ling et al., 2013; Hezroni et al., 2015; Ning et al., 2017).

The vast majority of NATs are expressed at low levels, one to three magnitudes lower than the corresponding sense transcripts, and the two RNAs tend to co-purify (Okazaki et al., 2002; Werner et al., 2007; Ling et al., 2013). In mammals, testis shows the highest level of antisense transcription, specifically in developing sperm cells (Carlile et al., 2009; Soumillon et al., 2013). However, the wide-spread antisense transcription in testis could be a mere consequence of the post-mitotic transcriptional burst during spermatogenesis (Lee et al., 2009; Laiho et al., 2013).

To what extent the sense and antisense transcripts are present in the same cell is often unclear. A recent report has demonstrated co-localization of Sox4 sense/antisense transcripts in mouse brain cells (Ling et al., 2016). Moreover, antisense transcripts in a head-to-head orientation tend to show concordant expression, possibly the result of bi-directional CpG-island containing promoters, which results in the co-expression of sense and antisense transcripts (Balbin et al., 2015). On the other hand, we and others have found limited evidence for the presence of genic dsRNA (Werner et al., 2014). It is conceivable, however, that co-expression of head-to-head sense/antisense pairs is tolerated whereas tail-to-tail pairs tend to exclude each other.

## Mechanisms of Antisense Regulation

There are three different levels at which bi-directional transcription and a putative NAT can affect the corresponding sense RNA. Firstly, transcription from one strand can interfere with the transcription on the opposite strand, thus influencing the production of the sense transcript, so-called ‘transcriptional interference’ (**Figure 2B**). This mechanism is often portrayed as two polymerase complexes crashing into each other which may happen under experimentally engineered circumstances but is unlikely to be relevant *in vivo* (Prescott and Proudfoot, 2002; Osato et al., 2007; Wang et al., 2014). A more likely cause of events would see transcription of one strand altering DNA structure and DNA–protein interactions of the specific locus on the opposite strand, thus affecting its transcription. Alternatively, two close transcription sites may compete for protein factors that enable initiation and elongation. Secondly, the complementary sense and antisense transcripts can hybridize and form a dsRNA intermediate (**Figure 2A**). This interaction potentially interferes with the processing of both RNAs, their splicing, nuclear export or even translation (Hastings et al., 2000; Ning et al., 2017). Alternatively, dsRNA is recognized by enzymes that resolve the double-strand structure and trigger various cellular responses (Wang and Carmichael, 2004). The best described dsRNA specific enzymes include ADARs (Adenosine Deaminases Acting on RNA) (Mannion et al., 2015), RNases type III (Dicer) (Svobodova et al., 2016) and protein kinase R (Munir and Berg, 2013). Thirdly, NATs may act independently of the cognate sense transcript and adopt the function of a long non-coding RNA. In fact, one of the best described lncRNA, HOTAIR, is transcribed antisense to HOXC11 and both HOTAIR and HOXC11 are concordantly upregulated in urothelial cancer (Heubach et al., 2015). Nevertheless, extensive research in the field has not investigated sense/antisense interactions but established HOTAIR’s interaction with polycomb repressive complex 2 and histone modification complexes (Heubach et al., 2015). The function of lncRNAs in sequestering proteins and miRNAs to provide a scaffold for regulatory complexes is described in detail elsewhere and is beyond the scope of this article (Rinn and Chang, 2012).

## Transcriptional Interference

It is well-established that expression of an antisense transcript leads to epigenetic repression of the related sense transcript. This has been extensively described in the context of X chromosome inactivation and parental imprinting. For example, suppression of Tsix (antisense to Xist) leads to ectopic expression of Xist and concomitant bi-allelic X inactivation in XX cells or silencing of the X chromosome in XY cells (Lee, 2000). Likewise, the imprinted gene clusters *Igf2r*/*Slc22a2*/*Slc22a3* and *Kcnq1* contain NATs (Airn and Kcnq1ot1, respectively) that are essential for parental imprinting (Sleutels et al., 2002; Thakur et al., 2004). Deletion of the antisense transcript interferes with the methylation status of the locus, alleviates silencing and leads to bi-allelic expression of the gene cluster (Sleutels et al., 2002; Mohammad et al., 2010). Thereby, the interference of the antisense transcript with either promoter or enhancer region triggers a gene-specific- or a broader response affecting the entire gene cluster, respectively (Kornienko et al., 2013). Bi-directional transcription is one of the key features of parentally imprinted gene clusters. The exact mechanistic consequences of the regulatory antisense transcripts, however, are not fully understood and distinct, cluster-specific differences occur (Kanduri, 2016).

In humans and mice, a few examples of transcriptional interference have been studied in detail and represent paradigms for the consequences of aberrant expression of NATs. All lead to specific pathological phenotypes that are related to the protein coding sense gene, predicting a strictly *cis*-acting mechanism of interaction. The first example relates to a rare form of α-thalassemia; in affected patients, the constitutively active LUC7L gene downstream of HBA2 is truncated, including the loss of the polyadenylation site. As a consequence, *LUC7L* transcription continues into *HBA2* and a NAT to *HBA2* is produced. Comparable effects were achieved when *LUC7L* was replaced with a different gene (*UBC*) confirming an essential role for transcription, independent of the nature of the gene (Tufarelli et al., 2003). The bi-directional tumor suppressor gene *p15*/*p15AS* shows a comparable arrangement, with a naturally occurring, lowly expressed antisense transcript (Yu et al., 2008). Enhanced expression of the antisense transcript and concomitant reduction of p15 cyclin-dependent kinase inhibitor was found in leukemia patient samples and also in two acute myeloid leukemia lines. The mechanism of silencing appeared to involve both altered histone modifications, increased H3K9me2 and decreased H3K4me2, as well as promoter DNA methylation depending on the cellular model system studied. Interestingly, a transfected construct that recapitulated the p15 genomic arrangement with inducible antisense transcription showed *cis*-silencing of the exogenous construct but also, with lesser penetrance, reduced endogenous p15. Both silencing mechanisms were shown to be Dicer-independent and to introduce stable epigenetic modifications (Yu et al., 2008). The reported *trans*-effect of the p15AS transcript suggests that the different mechanisms by which NATs interfere with sense transcript expression may depend on cell-specific features or sense/antisense transcript levels. Of note, transcriptional interference has also been reported between two consecutive genes on the same DNA strand. In a patient cohort with Lynch syndrome, the mismatch repair gene *MSH2* is epigenetically silenced by the truncated *TACSTD1 upstream* of *MSH2*. The resulting read-through transcript runs into *MSH2* and induces CpG methylation and silencing of the promoter (Ligtenberg et al., 2009). The underlying mechanism is yet unclear but could involve interactions of the read-through RNA with antisense transcripts produced from the bidirectional *MSH* promoter (Grzechnik et al., 2014; Uesaka et al., 2014).

## Double-Stranded RNA Formation

With the scale of antisense transcription emerging, it became evident that sense and antisense transcripts tend to be found in the same RNA preparations (Kiyosawa et al., 2003; Werner et al., 2007). This suggested that dsRNA formation could be an important intermediate in gene regulatory mechanisms involving NATs. The cellular pathways triggered by dsRNA were well-established at that time and included processing by Dicer into endo-siRNAs (RNA interference), A to I conversion by adenosine deaminases (RNA editing) as well as the activation of PKR (Wang and Carmichael, 2004). These pathways result in characteristic intermediates such as short RNAs, modified RNA or increased levels of IFN-α/β (Interferon), respectively, that are used as readouts for bi-directionally transcribed genes.

### RNA Interference

The initial discovery of RNA interference was based on the observation that introduction of dsRNA into *C. elegans* triggered highly specific, lasting gene knock-down (Fire et al., 1998). The strategies to adopt a similar approach in mammalian cells, however, failed almost completely. Only a few cell types, oocytes or certain embryonic cells, seem to tolerate significant levels of dsRNA without triggering an immune response (Wianny and Zernicka-Goetz, 2000; Piatek et al., 2017). As a consequence the contribution of Dicer and RNA interference to the processing of natural sense/antisense transcript pairs is controversial. There are a few reports that have identified short RNAs from endogenous RNA duplexes, so-called endo-siRNAs in vertebrates, predominantly in the germline and only few in somatic cells (Watanabe et al., 2008; Carlile et al., 2009; Xia et al., 2013; Werner et al., 2014; Jha et al., 2015). Nevertheless, the biological function of endo-siRNAs in the context of bi-directional transcription is not well-understood. Remarkably, however, both endo-siRNAs and NATs are predominantly found in mammalian testis in accordance with the proposed role in maintaining sperm genome integrity (Song et al., 2011; Soumillon et al., 2013; Werner et al., 2015).

### RNA Editing

Members of the ADAR family of adenosine deaminases recognize long stretches of dsRNA and convert adenosines into inosines. This process can either be site-specific or promiscuous (Mannion et al., 2015). Site-specific RNA editing is predominantly observed in the brain and affects a small number of neurotransmitter receptors. Since inosines pair with cytosines (rather than with thymidines) editing leads to point mutations and, consequently, to receptors with altered physiological properties (Schmauss and Howe, 2002). Promiscuous RNA editing acts on long stretches of dsRNA and involves widespread conversion of A to I. The modifications can resolve the double strand and/or interfere with nuclear export. RNA editing predominantly affects repetitive, intronic structures in a co-transcriptional process (Blow et al., 2004; Levanon et al., 2004). Both timing of hyper-editing and large-scale RNAseq data rule out a general contribution of RNA editing to natural sense/antisense RNA processing, though few gene specific regulatory mechanisms involving RNA editing have been reported (Prasanth et al., 2005; Salameh et al., 2015). Moreover, ADARs are induced by an antiviral interferon response and viral dsRNA was found to be hyper-edited supporting a role in innate immunity (Mannion et al., 2015).

### RNA Masking

An mRNA contains sequence motifs that are recognized by regulatory proteins and short RNA molecules to control its translation efficiency and half-life. Hybridization of an antisense transcript can potentially interfere with these regulatory interactions in a process called RNA masking. Both stabilizing and de-stabilizing effects of RNA masking by antisense transcripts have been reported. So far these include competition with miRNA binding sites (Faghihi et al., 2010; Liu et al., 2015) and exposure of mRNA degradation motifs (Cayre et al., 2003). In addition, a well-characterized example of antisense RNA-promoted alternative splicing has been reported (Hastings et al., 2000). Here, the levels of two alternative splice forms of the thyroid hormone receptor TRα1 and TRα2 correlate with the expression of an antisense transcript (RevErbα) complementary to the relevant splice site. RevErbα RNA sterically masks the TRα2-specific splice site and promotes TRα1 expression (Hastings et al., 1997). Interestingly, a genome-wide analysis of splicing events using a comprehensive set of exon array data found extensive correlation between antisense transcription and alternative exon usage (Morrissy et al., 2011). Moreover, genes with multiple splice forms are under-represented on metazoan sex chromosomes (Wegmann et al., 2008), a trend that mirrors the limited bi-directional transcription on mammalian X chromosomes (Kiyosawa et al., 2003; Chen et al., 2004). Somewhat counter intuitively, the apparent association between antisense transcription and alternative splicing has not been followed up and underpinned by examples of detailed analyses of specific loci.

Two well-documented examples of RNA masking focus on the bi-directionally transcribed genes BACE1 and HIF1α, both highly relevant to human disease, BACE1 in Alzheimer’s disease and HIF1α in cancer. In the former case, the antisense transcript BACE1-AS stabilizes the mRNA encoding β-secretase by masking the binding site of miR-485-5p (Faghihi et al., 2010). This leads to an increased production and accumulation of Amyloid-β (Faghihi et al., 2008). This particular example could apply to a number of bi-directionally transcribed genes since a significant number of NATs overlap with the 3′ end of the sense transcript (Faghihi et al., 2010). The second example of RNA masking includes the hypoxia-inducible factor 1α and the convergently transcribed antisense transcript aHIF (Rossignol et al., 2002). The antisense transcript is widely expressed in healthy tissues but significantly upregulated in various tumors and was proposed as a prognostic marker for cancer progression (Cayre et al., 2003; Dang et al., 2015). The inverse correlation between HIF1α and aHIF was hypothesized to result from an AU-rich element on the HIF1α RNA that becomes accessible upon antisense interaction.

### PKR and Innate Immunity

In a quick, first line response, the innate immune system reacts to specific bacterial and viral structures including glycans, lipopolysaccharides, particular proteins, and dsRNA. The latter is recognized by PKR that, upon binding to long RNA duplexes of >30 bp (Lemaire et al., 2008), undergoes dimerization and auto phosphorylation, reduces host protein synthesis and eventually triggers an interferon (IFN) response. Activation of IFN-α/β stimulates the expression of IFN inducible genes (including PKR), inhibits viral protein synthesis by phosphorylating eIF2-α and potentially triggers apoptosis (Marchal et al., 2014). Despite the fact that both PKR and Dicer process dsRNA in the cytoplasm, PKR activation and the IFN response prevail. This is also the reason why gene silencing by RNA interference (as established in *C. elegans*) is not applicable in most mammalian cells (Billy et al., 2001). From the viewpoint of natural antisense transcription, this poses a major conceptual dilemma: many of the proposed mechanisms established with particular bi-directionally transcribed genes involve cytoplasmic dsRNA intermediates with the potential to activate PKR.

## Conclusion

The very nature of natural antisense transcription is enigmatic, as large genomes of complex organisms could comfortably accommodate the relatively small number of genes without much interference. Moreover, convergent transcription and dsRNA cause various levels of cellular stress that may even lead to cell death. Nevertheless, NATs are abundant non-coding RNAs that potentially regulate their corresponding sense transcript through a variety of molecular mechanisms. Detailed research into the interplay of sense/antisense transcripts from specific loci has validated biological roles for antisense transcripts, yet mechanistic insights are still rare. As a consequence, the central question why bi-directionally transcribed loci persist and even expand during evolution is still unclear. A way forward here may link particular mechanisms (transcriptional interference, dsRNA formation and RNA interference, RNA masking, RNA editing) to specific categories of NATs (head-to-head, tail-to-tail) and assess these groups in model systems with or without the enzymatic components potentially involved in antisense RNA processing.

## Author Contributions

HZ, IN, and AW wrote the manuscript and designed the figures.

## Conflict of Interest Statement

The authors declare that the research was conducted in the absence of any commercial or financial relationships that could be construed as a potential conflict of interest.

## References

[B1] Abrusan G. Giordano J. Warburton P. E. (2008). Analysis of transposon interruptions suggests selection for L1 elements on the X chromosome. *PLOS Genet.* 4:e1000172. 10.1371/journal.pgen.1000172 18769724 PMC2517846

[B2] Balbin O. A. Malik R. Dhanasekaran S. M. Prensner J. R. Cao X. Wu Y. M. (2015). The landscape of antisense gene expression in human cancers. *Genome Res.* 25 1068–1079. 10.1101/gr.180596.114 26063736 PMC4484389

[B3] Baulcombe D. (2004). RNA silencing in plants. *Nature* 431 356–363. 10.1038/nature02874 15372043

[B4] Beiter T. Reich E. Williams R. W. Simon P. (2009). Antisense transcription: a critical look in both directions. *Cell Mol. Life Sci.* 66 94–112. 10.1007/s00018-008-8381-y 18791843 PMC11131530

[B5] Billy E. Brondani V. Zhang H. Muller U. Filipowicz W. (2001). Specific interference with gene expression induced by long, double-stranded RNA in mouse embryonal teratocarcinoma cell lines. *Proc. Natl. Acad. Sci. U.S.A.* 98 14428–14433. 10.1073/pnas.261562698 11724966 PMC64698

[B6] Blow M. Futreal P. A. Wooster R. Stratton M. R. (2004). A survey of RNA editing in human brain. *Genome Res.* 14 2379–2387. 10.1101/gr.2951204 15545495 PMC534661

[B7] Carlile M. Swan D. Jackson K. Preston-Fayers K. Ballester B. Flicek P. (2009). Strand selective generation of endo-siRNAs from the Na/phosphate transporter gene Slc34a1 in murine tissues. *Nucleic Acids Res.* 37 2274–2282. 10.1093/nar/gkp088 19237395 PMC2673434

[B8] Cayre A. Rossignol F. Clottes E. Penault-Llorca F. (2003). aHIF but not HIF-1alpha transcript is a poor prognostic marker in human breast cancer. *Breast Cancer Res.* 5 R223–R230. 10.1186/bcr652 14580258 PMC314412

[B9] Chan J. Atianand M. Jiang Z. Carpenter S. Aiello D. Elling R. (2015). Cutting edge: a natural antisense transcript, AS-IL1alpha, controls inducible transcription of the proinflammatory cytokine IL-1α. *J. Immunol.* 195 1359–1363. 10.4049/jimmunol.1500264 26179904 PMC4530055

[B10] Chen J. Sun M. Kent W. J. Huang X. Xie H. Wang W. (2004). Over 20% of human transcripts might form sense-antisense pairs. *Nucleic Acids Res.* 32 4812–4820. 10.1093/nar/gkh818 15356298 PMC519112

[B11] Chen L. Dahlstrom J. E. Lee S. H. Rangasamy D. (2012). Naturally occurring endo-siRNA silences LINE-1 retrotransposons in human cells through DNA methylation. *Epigenetics* 7 758–771. 10.4161/epi.20706 22647391

[B12] Conley A. B. Miller W. J. Jordan I. K. (2008). Human cis natural antisense transcripts initiated by transposable elements. *Trends Genet.* 24 53–56. 10.1016/j.tig.2007.11.008 18192066

[B13] Cullen B. R. Cherry S. Tenoever B. R. (2013). Is RNA interference a physiologically relevant innate antiviral immune response in mammals? *Cell Host Microbe* 14 374–378. 10.1016/j.chom.2013.09.011 24139396

[B14] Dang Y. Lan F. Ouyang X. Wang K. Lin Y. Yu Y. (2015). Expression and clinical significance of long non-coding RNA HNF1A-AS1 in human gastric cancer. *World J. Surg. Oncol.* 13 302. 10.1186/s12957-015-0706-3 26472090 PMC4608159

[B15] Diederichs S. (2014). The four dimensions of noncoding RNA conservation. *Trends Genet.* 30 121–123. 10.1016/j.tig.2014.01.004 24613441

[B16] Duharcourt S. Lepere G. Meyer E. (2009). Developmental genome rearrangements in ciliates: a natural genomic subtraction mediated by non-coding transcripts. *Trends Genet.* 25 344–350. 10.1016/j.tig.2009.05.007 19596481

[B17] Emerson J. J. Kaessmann H. Betran E. Long M. (2004). Extensive gene traffic on the mammalian X chromosome. *Science* 303 537–540. 10.1126/science.1090042 14739461

[B18] Faghihi M. A. Modarresi F. Khalil A. M. Wood D. E. Sahagan B. G. Morgan T. E. (2008). Expression of a noncoding RNA is elevated in Alzheimer’s disease and drives rapid feed-forward regulation of beta-secretase. *Nat. Med.* 14 723–730. 10.1038/nm1784 18587408 PMC2826895

[B19] Faghihi M. A. Zhang M. Huang J. Modarresi F. Van Der Brug M. P. Nalls M. A. (2010). Evidence for natural antisense transcript-mediated inhibition of microRNA function. *Genome Biol.* 11:R56. 10.1186/gb-2010-11-5-r56 20507594 PMC2898074

[B20] Fire A. Xu S. Montgomery M. K. Kostas S. A. Driver S. E. Mello C. C. (1998). Potent and specific genetic interference by double-stranded RNA in *Caenorhabditis elegans*. *Nature* 391 806–811. 10.1038/35888 9486653

[B21] Garcia-Lopez J. Hourcade Jde D. Alonso L. Cardenas D. B. Del Mazo J. (2014). Global characterization and target identification of piRNAs and endo-siRNAs in mouse gametes and zygotes. *Biochim. Biophys. Acta* 1839 463–475. 10.1016/j.bbagrm.2014.04.006 24769224

[B22] Goff L. A. Rinn J. L. (2015). Linking RNA biology to lncRNAs. *Genome Res.* 25 1456–1465. 10.1101/gr.191122.115 26430155 PMC4579330

[B23] Grzechnik P. Tan-Wong S. M. Proudfoot N. J. (2014). Terminate and make a loop: regulation of transcriptional directionality. *Trends Biochem. Sci.* 39 319–327. 10.1016/j.tibs.2014.05.001 24928762 PMC4085477

[B24] Gu W. Shirayama M. Conte D. Jr. Vasale J. Batista P. J. Claycomb J. M. (2009). Distinct argonaute-mediated 22G-RNA pathways direct genome surveillance in the *C. elegans* germline. *Mol. Cell* 36 231–244. 10.1016/j.molcel.2009.09.020 19800275 PMC2776052

[B25] Hastings M. L. Ingle H. A. Lazar M. A. Munroe S. H. (2000). Post-transcriptional regulation of thyroid hormone receptor expression by cis-acting sequences and a naturally occurring antisense RNA. *J. Biol. Chem.* 275 11507–11513. 10.1074/jbc.275.15.11507 10753970

[B26] Hastings M. L. Milcarek C. Martincic K. Peterson M. L. Munroe S. H. (1997). Expression of the thyroid hormone receptor gene, erbAalpha, in B lymphocytes: alternative mRNA processing is independent of differentiation but correlates with antisense RNA levels. *Nucleic Acids Res.* 25 4296–4300. 10.1093/nar/25.21.4296 9336460 PMC147039

[B27] Heubach J. Monsior J. Deenen R. Niegisch G. Szarvas T. Niedworok C. (2015). The long noncoding RNA HOTAIR has tissue and cell type-dependent effects on HOX gene expression and phenotype of urothelial cancer cells. *Mol. Cancer* 14:108. 10.1186/s12943-015-0371-8 25994132 PMC4455698

[B28] Hezroni H. Koppstein D. Schwartz M. G. Avrutin A. Bartel D. P. Ulitsky I. (2015). Principles of long noncoding RNA evolution derived from direct comparison of transcriptomes in 17 species. *Cell Rep.* 11 1110–1122. 10.1016/j.celrep.2015.04.023 25959816 PMC4576741

[B29] Ilott N. E. Heward J. A. Roux B. Tsitsiou E. Fenwick P. S. Lenzi L. (2014). Long non-coding RNAs and enhancer RNAs regulate the lipopolysaccharide-induced inflammatory response in human monocytes. *Nat. Commun.* 5:3979. 10.1038/ncomms4979 24909122 PMC4061460

[B30] Jeffrey K. L. Li Y. Ding S. W. (2017). Reply to ‘Questioning antiviral RNAi in mammals’. *Nat. Microbiol.* 2:17053. 10.1038/nmicrobiol.2017.53 28440274 PMC5488271

[B31] Jha A. Panzade G. Pandey R. Shankar R. (2015). A legion of potential regulatory sRNAs exists beyond the typical microRNAs microcosm. *Nucleic Acids Res.* 43 8713–8724. 10.1093/nar/gkv871 26354861 PMC4605316

[B32] Kanduri C. (2016). Long noncoding RNAs: lessons from genomic imprinting. *Biochim. Biophys. Acta* 1859 102–111. 10.1016/j.bbagrm.2015.05.006 26004516

[B33] Katayama S. Tomaru Y. Kasukawa T. Waki K. Nakanishi M. Nakamura M. (2005). Antisense transcription in the mammalian transcriptome. *Science* 309 1564–1566. 10.1126/science.1112009 16141073

[B34] Kiyosawa H. Yamanaka I. Osato N. Kondo S. Hayashizaki Y. (2003). Antisense transcripts with FANTOM2 clone set and their implications for gene regulation. *Genome Res.* 13 1324–1334. 10.1101/gr.982903 12819130 PMC403655

[B35] Korhonen H. M. Meikar O. Yadav R. P. Papaioannou M. D. Romero Y. Da Ros M. (2011). Dicer is required for haploid male germ cell differentiation in mice. *PLOS ONE* 6:e24821. 10.1371/journal.pone.0024821 21949761 PMC3174967

[B36] Kornienko A. E. Guenzl P. M. Barlow D. P. Pauler F. M. (2013). Gene regulation by the act of long non-coding RNA transcription. *BMC Biol.* 11:59. 10.1186/1741-7007-11-59 23721193 PMC3668284

[B37] Laiho A. Kotaja N. Gyenesei A. Sironen A. (2013). Transcriptome profiling of the murine testis during the first wave of spermatogenesis. *PLOS ONE* 8:e61558. 10.1371/journal.pone.0061558 23613874 PMC3629203

[B38] Lee J. T. (2000). Disruption of imprinted X inactivation by parent-of-origin effects at Tsix. *Cell* 103 17–27. 10.1016/S0092-8674(00)00101-X 11051544

[B39] Lee T. L. Pang A. L. Rennert O. M. Chan W. Y. (2009). Genomic landscape of developing male germ cells. *Birth Defects Res. C Embryo Today* 87 43–63. 10.1002/bdrc.20147 19306351 PMC2939912

[B40] Lehner B. Williams G. Campbell R. D. Sanderson C. M. (2002). Antisense transcripts in the human genome. *Trends Genet.* 18 63–65. 10.1016/S0168-9525(02)02598-2 11818131

[B41] Lemaire P. A. Anderson E. Lary J. Cole J. L. (2008). Mechanism of PKR Activation by dsRNA. *J. Mol. Biol.* 381 351–360. 10.1016/j.jmb.2008.05.056 18599071 PMC2570377

[B42] Levanon E. Y. Eisenberg E. Yelin R. Nemzer S. Hallegger M. Shemesh R. (2004). Systematic identification of abundant A-to-I editing sites in the human transcriptome. *Nat. Biotechnol.* 22 1001–1005. 10.1038/nbt996 15258596

[B43] Ligtenberg M. J. Kuiper R. P. Chan T. L. Goossens M. Hebeda K. M. Voorendt M. (2009). Heritable somatic methylation and inactivation of MSH2 in families with Lynch syndrome due to deletion of the 3′ exons of TACSTD1. *Nat. Genet.* 41 112–117. 10.1038/ng.283 19098912

[B44] Ling K. H. Brautigan P. J. Moore S. Fraser R. Cheah P. S. Raison J. M. (2016). Derivation of an endogenous small RNA from double-stranded Sox4 sense and natural antisense transcripts in the mouse brain. *Genomics* 107 88–99. 10.1016/j.ygeno.2016.01.006 26802803

[B45] Ling M. H. Ban Y. Wen H. Wang S. M. Ge S. X. (2013). Conserved expression of natural antisense transcripts in mammals. *BMC Genomics* 14:243. 10.1186/1471-2164-14-243 23577827 PMC3635984

[B46] Liu J. Wu W. Jin J. (2015). A novel mutation in SIRT1-AS leading to a decreased risk of HCC. *Oncol. Rep.* 34 2343–2350. 10.3892/or.2015.4205 26324025

[B47] Mannion N. Arieti F. Gallo A. Keegan L. P. O’connell M. A. (2015). New insights into the biological role of mammalian ADARs; the RNA editing proteins. *Biomolecules* 5 2338–2362. 10.3390/biom5042338 26437436 PMC4693238

[B48] Marchal J. A. Lopez G. J. Peran M. Comino A. Delgado J. R. Garcia-Garcia J. A. (2014). The impact of PKR activation: from neurodegeneration to cancer. *FASEB J.* 28 1965–1974. 10.1096/fj.13-248294 24522206

[B49] Mattick J. S. (2001). Non-coding RNAs: the architects of eukaryotic complexity. *EMBO Rep.* 2 986–991. 10.1093/embo-reports/kve230 11713189 PMC1084129

[B50] Matzke M. A. Birchler J. A. (2005). RNAi-mediated pathways in the nucleus. *Nat. Rev. Genet.* 6 24–35. 10.1038/nrg1500 15630419

[B51] Mohammad F. Mondal T. Guseva N. Pandey G. K. Kanduri C. (2010). Kcnq1ot1 noncoding RNA mediates transcriptional gene silencing by interacting with Dnmt1. *Development* 137 2493–2499. 10.1242/dev.048181 20573698

[B52] Morrissy A. S. Griffith M. Marra M. A. (2011). Extensive relationship between antisense transcription and alternative splicing in the human genome. *Genome Res.* 21 1203–1212. 10.1101/gr.113431.110 21719572 PMC3149488

[B53] Munir M. Berg M. (2013). The multiple faces of protein kinase R in antiviral defense. *Virulence* 4 85–89. 10.4161/viru.23134 23314571 PMC3544753

[B54] Ning Q. Li Y. Wang Z. Zhou S. Sun H. Yu G. (2017). The evolution and expression pattern of human overlapping lncRNA and protein-coding gene pairs. *Sci. Rep.* 7:42775. 10.1038/srep42775 28344339 PMC5366806

[B55] Okamura K. Lai E. C. (2008). Endogenous small interfering RNAs in animals. *Nat. Rev. Mol. Cell Biol.* 9 673–678. 10.1038/nrm2479 18719707 PMC2729316

[B56] Okazaki Y. Furuno M. Kasukawa T. Adachi J. Bono H. Kondo S. (2002). Analysis of the mouse transcriptome based on functional annotation of 60,770 full-length cDNAs. *Nature* 420 563–573. 10.1038/nature01266 12466851

[B57] Osato N. Suzuki Y. Ikeo K. Gojobori T. (2007). Transcriptional interferences in cis natural antisense transcripts of humans and mice. *Genetics* 176 1299–1306. 10.1534/genetics.106.069484 17409075 PMC1894591

[B58] Piatek M. J. Henderson V. Fearn A. Chaudhry B. Werner A. (2017). Ectopically expressed Slc34a2a sense-antisense transcripts cause a cerebellar phenotype in zebrafish embryos depending on RNA complementarity and Dicer. *PLOS ONE* 12:e0178219. 10.1371/journal.pone.0178219 28542524 PMC5436864

[B59] Prasanth K. V. Prasanth S. G. Xuan Z. Hearn S. Freier S. M. Bennett C. F. (2005). Regulating gene expression through RNA nuclear retention. *Cell* 123 249–263. 10.1016/j.cell.2005.08.033 16239143

[B60] Prescott E. M. Proudfoot N. J. (2002). Transcriptional collision between convergent genes in budding yeast. *Proc. Natl. Acad. Sci. U.S.A.* 99 8796–8801. 10.1073/pnas.132270899 12077310 PMC124378

[B61] Rinn J. L. Chang H. Y. (2012). Genome regulation by long noncoding RNAs. *Annu. Rev. Biochem.* 81 145–166. 10.1146/annurev-biochem-051410-092902 22663078 PMC3858397

[B62] Rossignol F. Vache C. Clottes E. (2002). Natural antisense transcripts of hypoxia-inducible factor 1α are detected in different normal and tumour human tissues. *Gene* 299 135–140. 10.1016/S0378-1119(02)01049-1 12459261

[B63] Salameh A. Lee A. K. Cardo-Vila M. Nunes D. N. Efstathiou E. Staquicini F. I. (2015). PRUNE2 is a human prostate cancer suppressor regulated by the intronic long noncoding RNA PCA3. *Proc. Natl. Acad. Sci. U.S.A.* 112 8403–8408. 10.1073/pnas.1507882112 26080435 PMC4500257

[B64] Schmauss C. Howe J. R. (2002). RNA editing of neurotransmitter receptors in the mammalian brain. *Sci. STKE* 2002:pe26. 10.1126/stke.2002.133.pe26 12023441

[B65] Shendure J. Church G. M. (2002). Computational discovery of sense-antisense transcription in the human and mouse genomes. *Genome Biol.* 3:RESEARCH0044. 10.1186/gb-2002-3-9-research0044 12225583 PMC126869

[B66] Sleutels F. Zwart R. Barlow D. P. (2002). The non-coding Air RNA is required for silencing autosomal imprinted genes. *Nature* 415 810–813. 10.1038/415810a 11845212

[B67] Song R. Hennig G. W. Wu Q. Jose C. Zheng H. Yan W. (2011). Male germ cells express abundant endogenous siRNAs. *Proc. Natl. Acad. Sci. U.S.A.* 108 13159–13164. 10.1073/pnas.1108567108 21788498 PMC3156200

[B68] Soumillon M. Necsulea A. Weier M. Brawand D. Zhang X. Gu H. (2013). Cellular source and mechanisms of high transcriptome complexity in the mammalian testis. *Cell Rep.* 3 2179–2190. 10.1016/j.celrep.2013.05.031 23791531

[B69] Stein P. Rozhkov N. V. Li F. Cardenas F. L. Davydenko O. Vandivier L. E. (2015). Essential Role for endogenous siRNAs during meiosis in mouse oocytes. *PLOS Genet.* 11:e1005013. 10.1371/journal.pgen.1005013 25695507 PMC4335007

[B70] Svobodova E. Kubikova J. Svoboda P. (2016). Production of small RNAs by mammalian Dicer. *Pflugers Arch.* 468 1089–1102. 10.1007/s00424-016-1817-6 27048428 PMC4893058

[B71] tenOever B. R. (2017). Questioning antiviral RNAi in mammals. *Nat. Microbiol.* 2:17052. 10.1038/nmicrobiol.2017.52 28440277

[B72] Thakur N. Tiwari V. K. Thomassin H. Pandey R. R. Kanduri M. Gondor A. (2004). An antisense RNA regulates the bidirectional silencing property of the Kcnq1 imprinting control region. *Mol. Cell. Biol.* 24 7855–7862. 10.1128/MCB.24.18.7855-7862.2004 15340049 PMC515059

[B73] Tufarelli C. Stanley J. A. Garrick D. Sharpe J. A. Ayyub H. Wood W. G. (2003). Transcription of antisense RNA leading to gene silencing and methylation as a novel cause of human genetic disease. *Nat. Genet.* 34 157–165. 10.1038/ng1157 12730694

[B74] Uesaka M. Nishimura O. Go Y. Nakashima K. Agata K. Imamura T. (2014). Bidirectional promoters are the major source of gene activation-associated non-coding RNAs in mammals. *BMC Genomics* 15:35. 10.1186/1471-2164-15-35 24438357 PMC3898825

[B75] Veeramachaneni V. Makalowski W. Galdzicki M. Sood R. Makalowska I. (2004). Mammalian overlapping genes: the comparative perspective. *Genome Res.* 14 280–286. 10.1101/gr.1590904 14762064 PMC327103

[B76] Verona R. I. Mann M. R. Bartolomei M. S. (2003). Genomic imprinting: intricacies of epigenetic regulation in clusters. *Annu. Rev. Cell Dev. Biol.* 19 237–259. 10.1146/annurev.cellbio.19.111401.092717 14570570

[B77] Wang L. Jiang N. Wang L. Fang O. Leach L. J. Hu X. (2014). 3′ Untranslated regions mediate transcriptional interference between convergent genes both locally and ectopically in *Saccharomyces cerevisiae*. *PLOS Genet.* 10:e1004021. 10.1371/journal.pgen.1004021 24465217 PMC3900390

[B78] Wang Q. Carmichael G. G. (2004). Effects of length and location on the cellular response to double-stranded RNA. *Microbiol. Mol. Biol. Rev.* 68 432–452. 10.1128/MMBR.68.3.432-452.2004 15353564 PMC515255

[B79] Watanabe T. Totoki Y. Toyoda A. Kaneda M. Kuramochi-Miyagawa S. Obata Y. (2008). Endogenous siRNAs from naturally formed dsRNAs regulate transcripts in mouse oocytes. *Nature* 453 539–543. 10.1038/nature06908 18404146

[B80] Wegmann D. Dupanloup I. Excoffier L. (2008). Width of gene expression profile drives alternative splicing. *PLOS ONE* 3:e3587. 10.1371/journal.pone.0003587 18974852 PMC2575406

[B81] Weinberg M. S. Morris K. V. (2016). Transcriptional gene silencing in humans. *Nucleic Acids Res.* 44 6505–6517. 10.1093/nar/gkw139 27060137 PMC5001580

[B82] Werner A. Carlile M. Swan D. (2009). What do natural antisense transcripts regulate? *RNA Biol.* 6 43–48. 19098462 10.4161/rna.6.1.7568

[B83] Werner A. Cockell S. Falconer J. Carlile M. Alnumeir S. Robinson J. (2014). Contribution of natural antisense transcription to an endogenous siRNA signature in human cells. *BMC Genomics* 15:19. 10.1186/1471-2164-15-19 24410956 PMC3898206

[B84] Werner A. Piatek M. J. Mattick J. S. (2015). Transpositional shuffling and quality control in male germ cells to enhance evolution of complex organisms. *Ann. N. Y. Acad. Sci.* 1341 156–163. 10.1111/nyas.12608 25557795 PMC4390386

[B85] Werner A. Schmutzler G. Carlile M. Miles C. G. Peters H. (2007). Expression profiling of antisense transcripts on DNA arrays. *Physiol. Genomics* 28 294–300. 10.1152/physiolgenomics.00127.2006 17105753

[B86] Werner A. Swan D. (2010). What are natural antisense transcripts good for? *Biochem. Soc. Trans.* 38 1144–1149. 10.1042/BST0381144 20659019 PMC4284956

[B87] Wianny F. Zernicka-Goetz M. (2000). Specific interference with gene function by double-stranded RNA in early mouse development. *Nat. Cell Biol.* 2 70–75. 10.1038/35000016 10655585

[B88] Wight M. Werner A. (2013). The functions of natural antisense transcripts. *Essays Biochem.* 54 91–101. 10.1042/bse0540091 23829529 PMC4284957

[B89] Wood E. J. Chin-Inmanu K. Jia H. Lipovich L. (2013). Sense-antisense gene pairs: sequence, transcription, and structure are not conserved between human and mouse. *Front. Genet.* 4:183 10.3389/fgene.2013.00183 PMC3783845 24133500

[B90] Wyler E. Menegatti J. Franke V. Kocks C. Boltengagen A. Hennig T. (2017). Widespread activation of antisense transcription of the host genome during herpes simplex virus 1 infection. *Genome Biol.* 18:209. 10.1186/s13059-017-1329-5 29089033 PMC5663069

[B91] Xia J. Joyce C. E. Bowcock A. M. Zhang W. (2013). Noncanonical microRNAs and endogenous siRNAs in normal and psoriatic human skin. *Hum. Mol. Genet.* 22 737–748. 10.1093/hmg/dds481 23175445 PMC3554200

[B92] Yu W. Gius D. Onyango P. Muldoon-Jacobs K. Karp J. Feinberg A. P. (2008). Epigenetic silencing of tumour suppressor gene p15 by its antisense RNA. *Nature* 451 202–206. 10.1038/nature06468 18185590 PMC2743558

